# Optimization of a High-Throughput Human Papillomavirus Neutralizing Antibody Assay Based on Pseudotyped Viruses for the 15-Valent Human Papillomavirus Vaccine Types

**DOI:** 10.3390/v17091164

**Published:** 2025-08-26

**Authors:** Huan Liu, Haiyang Qin, Lingling Nie, Yanru Shen, Jiayi Li, Pengcheng Xiu, Shasha Wang, Meng Wang, Youchun Wang, Jianhui Nie, Weijin Huang, Li Zhang

**Affiliations:** 1 Division of HIV/AIDS and Sex-Transmitted Virus Vaccines, Institute for Biological Product Control, National Institutes for Food and Drug Control (NIFDC), Beijing 102629, China; lh3253936@163.com (H.L.); qinhaiyang@nifdc.org.cn (H.Q.);; 2 Hebei Key Laboratory of Public Health Safety, School of Public Health, Hebei University, Baoding 071000, China; 3 National Laboratory Animal Quality Testing Center, Institute of Laboratory Animal Resources, National Institutes for Food and Drug Control, Beijing 102629, China; 4 Institute of Medical Biology, Chinese Academy of Medical Sciences and Peking Union Medical College, Kunming 650118, China

**Keywords:** human papillomavirus, pseudovirion-based neutralization assay (PBNA), immunogenicity, high-throughput assay

## Abstract

Vaccination is highly effective in preventing human papillomavirus (HPV) infection, but traditional pseudovirion-based neutralization assays (PBNA) are technically demanding, labor-intensive, and costly, limiting their use in multivalent vaccine studies. We developed and validated an automated, high-throughput PBNA in a 384-well format that quantifies neutralizing antibodies against 15 HPV types using triple-color pseudotyped viruses. Non-interfering type triplets were defined from cross-neutralization assays of serum against pseudotyped viruses, enabling simultaneous detection of three fluorescence signals per well. The workflow integrates a cap-decapper, semi-automatic sample addition and dilution, and a microplate stacker with automated imaging to reduce hands-on time. The 384-well method showed strong concordance with the conventional 96-well PBNA while increasing daily sample throughput by approximately 6.7-fold, reducing assay duration (including ~4-fold faster imaging), and lowering reaction volume by ~5-fold. Analytical validation demonstrated acceptable specificity, accuracy, repeatability, linearity and robustness for high-throughput use. Serostatus cutoff values were established in an age-appropriate female population to support classification of positive versus negative sera. This platform provides a scalable tool for evaluating neutralizing antibodies after natural infections or vaccination and is well suited for large clinical trials and the development of next-generation and multivalent HPV vaccines.

## 1. Introduction

Globally, cervical cancer is the fourth most common cause of cancer incidence and mortality among women, with an estimated 660,000 new cases and about 350,000 deaths each year [1]. Human papillomavirus (HPV) is a non-enveloped, double-stranded DNA virus (family Papillomaviridae) and the necessary cause of virtually all cervical cancers [2]; more than 200 HPV types have been described [3,4], with a subset of oncogenic types linked to cervical, anal, and oropharyngeal cancers, while others primarily cause benign lesions such as genital warts [5]. Prophylactic vaccines targeting HPV16/18 (bivalent), HPV6/11/16/18 (quadrivalent), and nine types including 6/11/16/18/31/33/45/52/58 (nonavalent) have markedly reduced vaccine-type infections and related disease [6], and next-generation multivalent candidates (e.g., 11-, 14-, and 15-valent formulations) aim to broaden coverage further [7].

Assessing the immunogenicity of these vaccines relies on functional antibody measurements. While pivotal randomized HPV vaccine trials demonstrated efficacy by showing a significant reduction in CIN2+ incidence among vaccinated individuals compared to placebo controls, neutralizing antibodies remain a widely accepted surrogate marker of protection in clinical trials [8,9]. The World Health Organization (WHO) specifically recommends neutralizing antibody assays as the gold standard for assessing vaccine-induced protective responses [10]. However, due to the difficulties of HPV cultivating in vitro, pseudovirion-based neutralization assays (PBNA) are widely used to quantify neutralizing antibodies.

The procedures of HPV-specific PBNA include co-transfecting mammalian cells with reporter plasmids by utilizing two late capsid genes of HPV, L1 and L2. This process produces high-infectivity pseudotyped virus particles that possess surface conformational epitopes similar to those of natural viral particles-alike [11,12]. Various types of PBNAs have been developed based on different reporter genes, including chemiluminescent reporter genes (e.g., secreted alkaline phosphatase (SEAP) [13], Gaussia luciferase (Gluc) [14,15]) and multicolor fluorescent protein genes (EGFP, RFP, or E2-CFP) [16]. However, the PBNA also has some disadvantages, including being operationally complex, having a long detection cycle, low throughput, and high cost. Additionally, manual operation was also predominantly utilized during the detection phase. As the development of vaccines is increasingly targeting more HPV genotypes, the importance of detecting immunogenicity of multivalent HPV vaccines is growing. When using PBNA for immunogenicity testing on a large number of clinical samples, it is necessary to improve the automation and high-throughput capability of sample processing to reduce costs and improve efficiency.

Here, we optimize and validate an automated, high-throughput PBNA in a 384-well format that uses triple-color pseudotyped viruses to measure neutralizing antibodies against 15 HPV types. By selecting non-interfering type triplets and integrating semi-automated liquid handling with automated fluorescence readout, the assay reduces sample volume and assay time while enabling simultaneous detection of three types per well. We also establish serostatus cutoffs in an age-appropriate population, supporting applications in large-scale clinical trials and the development of multivalent HPV vaccines.

## 2. Materials and Methods

### 2.1. Cells, Serum, and Plasmids

We used 293FT cells (Invitrogen, R70007) for pseudotyped virus production and cell-based neutralization antibody detection assays, maintained in Dulbecco’s Modified Eagle Medium (DMEM) and supplemented with 10% fetal bovine serum (FBS, Pansera ES, PAN-Biotech), glutamine solution (GIBCO), non-essential amino acids (GIBCO) and penicillin/streptomycin (PS, GIBCO). All cells were incubated at 37 °C with 5% CO_2_.

New Zealand white rabbits (2.8–3.0 kg of weight, female) were subcutaneously injected with 100 μg monovalent HPV L1 virus-like particles (VLPs) with the adjuvant of aluminum hydroxide and 15-valent HPV vaccine candidate. Immunization was repeated three times with a 4-week interval, and serum was obtained 1 day before immunization and 3 weeks after final immunization. The monovalent vaccines were sourced from three candidate vaccines, Beijing Health Guard Biotechnology Inc. (Beijing, China), Sinocelltech Ltd. (Beijing, China), Chengdu Institute of Biological Products Co., Ltd (Chengdu, Sichuan Province, China). The 15-valent HPV vaccine candidate was from Beijing Health Guard Biotechnology Inc (Beijing, China).

The 57 positive serum samples were collected after the administrations of three doses of the nonavalent vaccine candidate (Shanghai Bovax Biotechnology Co., Ltd., Shanghai, China) and 200 samples of negative serum from women with aged ranged 16 to 26 were collected by the Zhejiang Provincial Center For Disease Control and Prevention.

The HPV L1/L2 expressing plasmids and the red fluorescent protein (RFP) reporter plasmid pRwB were kindly provided by John Schiller (National Cancer Institute, Bethesda, MD, USA). E2-CFP (Clontech, 632554) expresses the E2-crimson fluorescent protein (CFP). The EGFP reporter plasmid was constructed by inserting the EGFP gene into the pCDNA3.1 vector.

### 2.2. Preparation and Titration of HPV Pseudotyped Virus

#### 2.2.1. Production of Pseudotyped Virus

HPV pseudotyped virus was constructed in 293FT cells as described in previous studies [15,16]. HPV L1/L2 expression plasmids were co-transfected with a reporter plasmid in 293 FT cells using Lipofectamine 3000 (Invitrogen) for 4–6 h. Cells were collected 72 h after transfection and resuspended in Dulbecco’s Phosphate Buffered Saline (Gibco) supplemented with 0.5% Triton X-100, 0.1% Benzonase^®^ (Sigma-Aldrich, Merck KGaA, Darmstadt, Germany), 0.1% Plasmid-Safe™ ATP-Dependent DNase (Lucigen, Middleton, WI, USA), 4.0% ATP Buffer (Lucigen), 2.5% 1 M ammonium sulfate. Cell lysate was incubated at 37 °C overnight until the maturation of the pseudotyped virus. The matured lysate was then chilled on ice for 5 min and 0.17 volume of 5 M NaCl was added to adjust the salt concentration up to 850 mM. Following a 20 min incubation on ice, supernatant from the lysate was separated by centrifugation at a speed of 8000× *g*, in a refrigerated microcentrifuge for 10 min. The pseudotyped virus was subsequently aliquoted and frozen at −80 °C.

#### 2.2.2. Titration

Pseudotyped virus from each tube was prediluted by 1000-fold in 96-well U-shaped plates, then a 2-fold serial dilution was performed to obtain a total of seven different dilution multiples. Subsequently, sterile water (70 µL per well) was added to the edges of the 384-well plates to prevent the edge effects caused by internal liquid evaporation. Next, the diluted liquid was transferred from the 96-well U-shaped plates to the 384-well plates using a liquid handling workstation (VIAFLO, INTEGRA Biosciences, Zizers, Switzerland). Next, 293 FT cells (3 × 10^3^, 20 µL) were added to each well automatically by the liquid handling workstation, and incubated at 37 °C and 5% CO_2_ for 60–96 h. Finally, the number of positive cells of different fluorescent proteins was counted using the ImmunoSpot reader (Biotek Cytation 5, Agilent Technologies, Winooski, VT, USA), and the titer of the HPV pseudotyped viruses was calculated based on the fluorescent counts.

### 2.3. Pseudovirion-Based Neutralization Assay

#### 2.3.1. Traditional 96-Well Plate Method

The 293FT cells were seeded in a 96-well cell culture plate at a density of 1.5 × 10^4^ cells/well and incubated at 37 °C and 5% CO_2_ for 4–8 h. Serum samples were diluted in a 96-well U-shaped plate (60 μL/well), and mixed with diluted pseudotyped viruses. After incubation at 4 °C for 1 h, the virus–serum mixture was transferred to the pre-inoculated 293FT cells, and incubated at 37 °C and 5% CO_2_ for 60–96 h. Fluorescent spots were quantified using ELISPOT ImmunoSpot reader (CTL).

#### 2.3.2. High-Throughput 384-Well Plate Method

**Figure 1:**
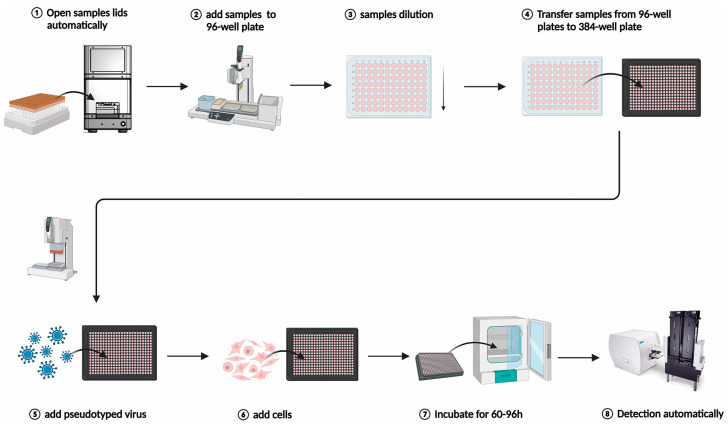
Establishment of an automatic neutralization test for HPV pseudotyped virus in a 384-well plate. This figure was drawn using Biorender. **1**. The lids of serum samples are opened by using the Micronic Screw Cap Recapper. **2**. The workstation aspirates the samples and adds them to A2-A11 wells of a 96-well plate. **3**. The workstation automatically dilutes and mixes the samples in a series from row A to G. **4**. Different dilutions of liquid (10 μL) from each sample are transferred from each sample in a 96-well plate to two corresponding replicate wells in a 384-well plate. Steps 2, 3 and 4 are efficiently completed through the INTEGRA ASSIST PLUS pipetting workstation. **5**. The diluted pseudotyped virus solution is dispensed into each well of a 384-well plate, with a volume of 10 μL per well. **6**. The cell diluted solution are aspirated and dispensed in the volume of 20 µL per well to the 384-well plate. Steps 5 and 6 are completed through the INTEGRA VIAFLO 384 multichannel handheld pipette. **7**. The cell plates are incubated in a 37 °C, 5% CO_2_ incubator for 60–72 h. **8**. The cultured cell plates are placed into BioStack Microplate Stacker 4 (Agilent Technologies, Winooski, VT, USA), and Cytation 5 is used for automated high-throughput fluorescence point imaging and quantitative analysis.

In this high-throughput 384-well plate neutralization assay, the dilution and addition of samples, cells, and pseudotyped virus, as well as the detection of fluorescent spots, are integrated into an automated process (Figure 1). The caps of sample tubes used for the neutralization assay were opened in an automatic capping machine. Subsequently, the samples were added to wells A2-A11 of a 96-well U-bottom plate using the INTEGRA ASSIST PLUS pipetting workstation and serum samples were subjected to a series of dilutions. In total, 10 μL of each diluted serum was transferred from the dilution plate to a 384-well plate. The pseudotyped virus stock was diluted to a concentration of 400 TCID_50_/mL, and 10 μL of the diluted pseudotyped virus was added to each well of the 384-well plate. Subsequently, the cell concentration was adjusted to 1.5 × 10^5^ cells/mL, with 20 μL of cells added to each well. The cell culture plates were then incubated at 37 °C with 5% CO_2_ for 60–96 h. In addition, untreated cells as negative control and cells treated only with pseudotyped virus as positive control. The ImmunoSpot reader was used to scan and count the number of fluorescent spots in each well. The neutralization titer of the sample is determined using the Reed–Muench method, expressed as the 50% maximal inhibitory dose (ID_50_), which is the reciprocal of the dilution factor that inhibits 50% of pseudotyped virus infection.

### 2.4. Statistical Analysis

The Pearson correlation coefficient was used to analyze the strength of linear relationships; the correlation coefficients should meet the predefined acceptance criterion (r ≥ 0.90). To evaluate the establishment and validation parameters, the Geometric Coefficient of Variation (GCV) was determined. The acceptance criterion requires that the GCV values from each independent condition should be ≤50%. The analysis of variance (ANOVA) test was used to assess the difference in quantitative results obtained from triple-type and single-type pseudotyped virus comparative assay, as well as the difference in quantitative results obtained from high-throughput PBNA and traditional PBNA. To evaluate the intra-assay variability and the inter-assay variability, the acceptance criterion requires that the Coefficient of Variation (CV) values from each independent condition should be ≤50%. The data were analyzed with SPSS V25.0 (Statistical Package for Social Science V.25.0) and GraphPad Prism 8.0 software (GraphPad, San Diego, CA, USA). The presentation of the results includes means ± standard deviation (SD). Significance thresholds: * *p* value less than 0.05, ** *p* value less than 0.01.

## 3. Results

### 3.1. Selection of Pseudotyped Virus Combinations with Three Types

**Figure 2:**
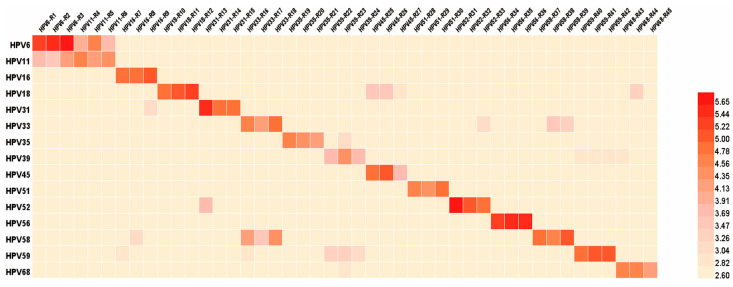
Heatmap of type-specific antibodies and cross-reactive antibodies titers. In this experiment, 15 types of HPV pseudotyped viruses were used to detect 15 positive rabbit sera after monovalent immunization. Serum samples were diluted at an initial dilution ratio of 400 times, and then continuously diluted at a gradient of four times per step to obtain neutralizing antibody titers.

In order to improve the throughput, the method based on the triple-color PBNA developed previously was optimized [16]. Considering the possibility of immunogenic cross-reactivity between different HPV viruses [17], the reactivity among 15 pseudotyped HPV was studied to find out the interference between those types. Initially, high titer monovalent serum with type-specific neutralizing antibodies was obtained by immunizing rabbits with monovalent L1 VLPs of 15 HPV. The median of neutralizing antibody titer in the monovalent sera for each type was 71,384 (IQR 35,802–15,8963; N = 45) (Table S1). Subsequently, the PBNA were performed using monovalent sera against non-specific types of pseudotyped viruses. Some monovalent rabbit sera can induce certain cross-neutralization effects with non-specific types of pseudotyped viruses, and have consistency in the same group of two or more monovalent rabbit sera (Figure 2). For example, when using pseudotyped HPV58 to detect the HPV33-positive serum, the geometric mean titer of cross-neutralizing antibodies was 11,637. The same situation also occurred between pseudotyped HPV18 and HPV45 positive serum—the value was 2283. Based on the results (Table S1), 15 pseudotyped HPV were divided into five groups: HPV6-33-45, HPV31-11-58, HPV16-18-68, HPV39-51-35, and HPV59-56-52, to ensure no cross-reactivity between HPV types in the same group and avoid non-specific type antibody values. Within each group, the three HPV types were labeled with green (GFP), red (RFP), and crimson (CFP) fluorescent proteins, respectively, with no cross-type interference (Table 1).

### 3.2. Comparison Between Triple-Type and Single-Type Pseudotyped Virus Detection

**Figure 3:**
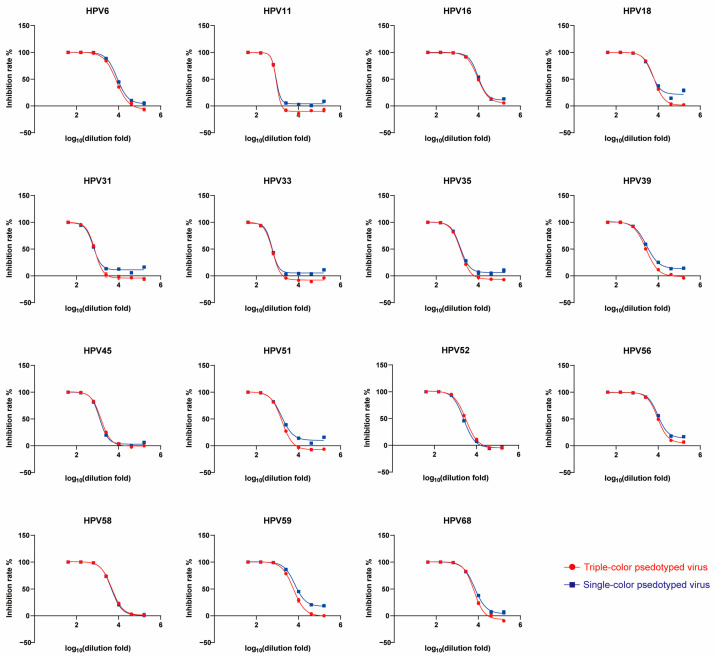
Comparison of triple-type and single-type pseudotyped virus in detecting inhibition rates. The graph shows the dilution fold and corresponding inhibition rate using triple-type and single-type pseudotyped virus. The red circle represents the inhibition rates against the triple-type pseudotyped virus, and the blue square represents the inhibition rates against the corresponding single-type pseudotyped virus. The horizontal axis shows the logarithmic dilutions of sera, while the vertical axis indicates the corresponding inhibition rates (%).

To determine whether the presence of three-HPV pseudotyped virus affects the high-throughput PBNA results, the same serum sample was tested by triple-type virus combinations and single-type virus, respectively. It was shown that the inhibition rates of the serum against the same virus exhibited similar typical four-parameter logistic inhibition rate curves. The curves indicated that there was no distinct difference between the two assays (R^2^ ≥ 0.90) (Figure 3). The ID_50_ values obtained from both detection assays showed a good correlation, with r ≥ 0.90 (0.90–0.99) (Table S2). There was also no statistically significant difference in the ID_50_ values of each type between triple-type virus combinations and single-type virus detection assays (*p* > 0.05) (Table S2), indicating good consistency between the two assays.

### 3.3. Establishment of High-Throughput Neutralization Assay Based on the Three-Type HPV Pseudotyped Virus

#### 3.3.1. Cell Concentration

**Figure 4:**
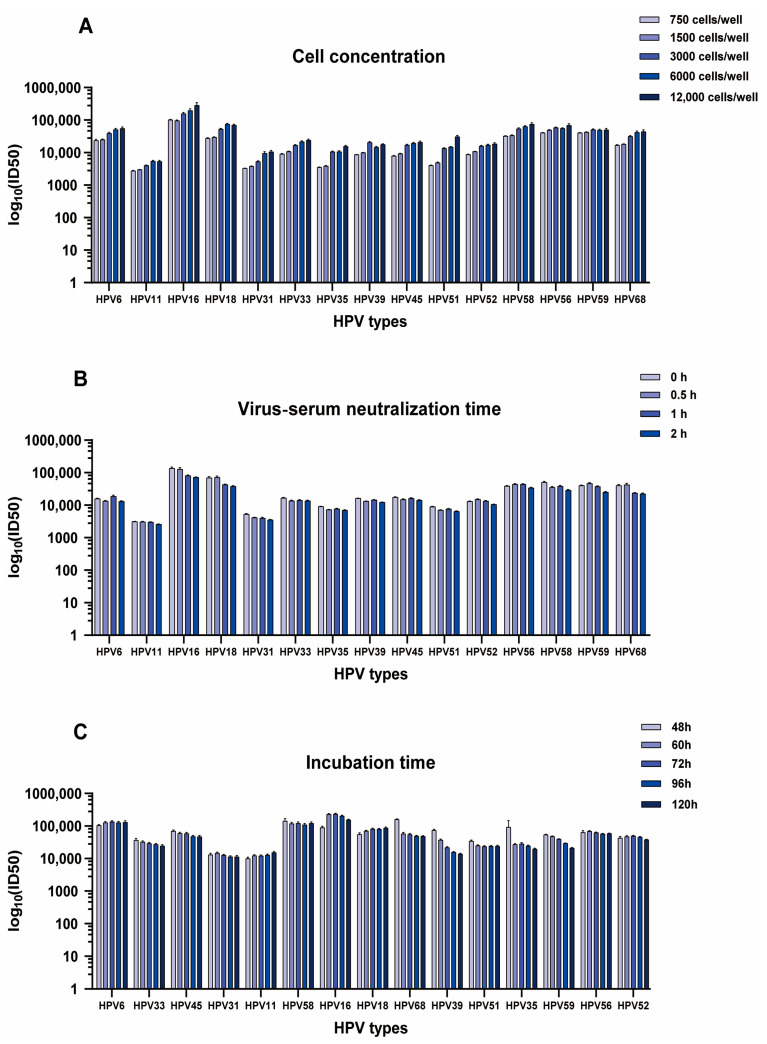
Optimization of the method for PBNA in a 384-well plate format. The graph illustrates the robustness of the assay across various experimental parameters, including cell concentration (**A**), virus-serum neutralization time (**B**), and incubation time (**C**). The horizontal axis indicates the HPV types, while the vertical axis indicates the neutralizing titers (mean ± standard deviation).

To determine the ideal concentration of 293 FT cells for use in the PBNA, the values of a 15-valent post-vaccination serum sample (15v antibody serum) were measured. The HPV neutralization antibody titers were then compared at cell concentrations of 750 cells/well, 1500 cells/well, 3000 cells/well, 6000 cells/well, and 12,000 cells/well. Following increased cell concentration, the ID_50_ values showed a gradual elevation, and the values obtained from 3000 to 12,000 cells/well were higher than those obtained from 750 to 1500 cells/well (Figure 4A). The GCV ranges of 750, 1500, 3000, 6000, and 12,000 cells/well were 4.3–26.1%, 4.8–17.1%, 10.9–40.4%, 13.7–60.4% and 20.4–119.4%, respectively (Table 2). Considering the assay values of the samples and the GCV criteria, 3000 cells per well were chosen as the experimental cell addition.

#### 3.3.2. Virus-Serum Neutralization Time

To determine the optimal pre-incubation time, cells were incubated with five combinations of HPV pseudotyped virus separately for 0 h, 0.5 h, 1 h, and 2 h to study the impacts of various pre-incubation durations on PBNA. Results demonstrated that the standard samples have exhibited a relatively low variability in neutralizing titer against five combinations of HPV pseudotyped virus despite the alternation of incubation time (GCV: 3.6–46.7%) (Table 2). Therefore, the incubation time has a minimal impact on the detection outcome, and the virus-serum neutralization time can be adjusted within a range of 0 h to 2 h (Figure 4B).

#### 3.3.3. Incubation Time

To determine the optimal detection time, the number of fluorescent positive cells at 24, 48, 60, 72, 96, and 120 h post co-culture of the serum pseudotyped virus mixture with cells were assessed. After 24 h of incubation, almost no fluorescent positive cells were observed. When detected after 48 h of incubation and beyond, the number of fluorescent positive cells gradually increased. It was shown that the sample ID_50_ values at 60–96 h (GCV: 5.2–43.8%) were relatively stable (Table 2). We chose to count the number of fluorescent positive cells between 60 and 96 h (Figure 4C).

### 3.4. Validation of High-Throughput Neutralization Assay Based on the Three-Type HPV Pseudotyped Virus

#### 3.4.1. Specificity

**Figure 5:**
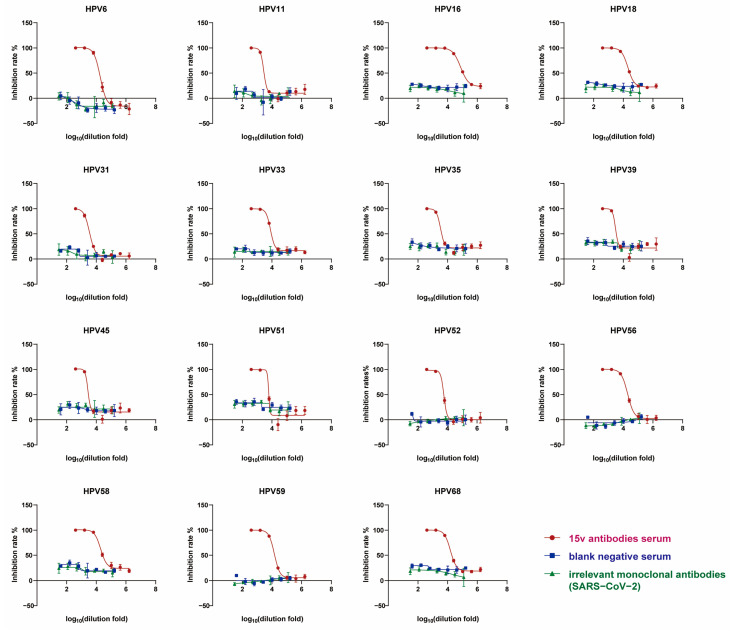
Specificity: rabbit serum immunized with a 15-valent vaccine (15v antibody serum) was subjected to neutralization tests against five combinations of HPV pseudotyped virus. Negative serum and irrelevant monoclonal antibodies (SARS-CoV-2) were used as controls in the neutralization tests. The red circle represents the rabbit serum immunized with a 15-valent vaccine, the blue square represents negative serum, and the green triangle represents the irrelevant monoclonal antibodies (SARS-CoV-2). The horizontal axis shows the logarithmic dilutions of sera, while the vertical axis indicates the corresponding inhibition rates (%).

Neutralization assays were performed using serially diluted antisera from rabbits immunized with the 15-valent vaccine against five combinations of HPV pseudotyped virus types. The results demonstrated characteristic sigmoidal dose–response curves with a goodness of fit (R^2^ ≥ 0.90) (Figure 5). In contrast, neither the negative control serum (from non-immunized rabbits) nor irrelevant pathogen-derived monoclonal antibodies (e.g., SARS-CoV-2) exhibited significant dose–response relationships under identical experimental conditions. Furthermore, when the assay was conducted with SARS-CoV-2 pseudotyped virus, only the SARS-CoV-2 monoclonal antibodies displayed a dose-dependent neutralization effect, whereas neither the 15-valent vaccine antisera nor the negative control serum showed detectable activity (Figure S1). These findings collectively indicate that the high-throughput neutralization assay exhibits excellent specificity, with no cross-reactivity observed from non-specific serum components, irrelevant pathogen-derived antibodies, or unrelated pseudotyped viruses.

#### 3.4.2. Serostatus Cutoff

In previous studies, the determination of the cutoff value for human serum neutralizing antibody titers was predicated on the analysis of 20 seronegative samples derived from infants aged 12 months. This assessment utilized pseudotyped viruses corresponding to HPV6, HPV11, HPV16, and HPV18, in conjunction with the non-related virus Bovine papillomavirus (BPV) as a control. The cutoff value of the method was set to 40; meanwhile, when the serum ID_50_ was greater than 40 and the value was greater than twice the BPV ID_50_, the sample was judged as positive [18]. Nevertheless, there are certain limitations to this approach for establishing cutoff, particularly concerning the limited size of the sample and the relatively youthful demographic of the participants involved.

To obtain a cutoff for neutralization tests accurately, 200 negative serum samples from females aged 16 to 26 were subjected to three repeated tests in this study. The negative sera samples were initially diluted 10-fold, followed by 2-fold serial dilutions to calculate ID_50_. A certain level of non-specific inhibition was produced at the low serum dilution against all 15 HPV types, with an average ID_50_ 20 to 54. The cutoff values of each HPV type were initially set to 48–177 (mean + 3 SD), and the positive rate for detecting 15 types of pseudotyped viruses was calculated between 1.0% and 3.6%, indicating a perfect method specificity (Table 2).

#### 3.4.3. Linearity

**Figure 6:**
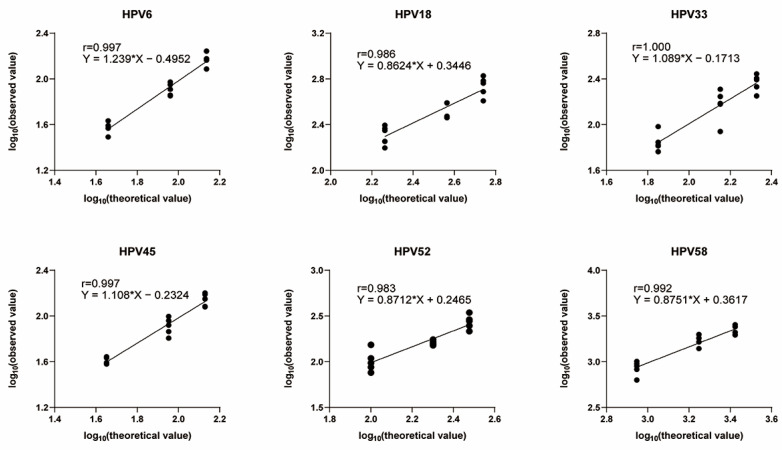
Linearity: standard antibodies for HPV6, HPV18, HPV33, HPV45, HPV52, and HPV58 produced by National Institutes for Food and Drug Control were subjected to neutralization tests against corresponding HPV pseudotyped virus. The r value indicates the correlation between the logarithmic theoretical and measured ID_50_ values. The regression equation demonstrates the linearity of the two methods. The horizontal axis shows the logarithmic theoretical ID_50_ of the sera, while the vertical axis indicates the corresponding logarithmic measured ID_50_. The asterisk (*) denotes multiplication. The black point on the fitted line represents logarithmic theoretical ID_50_ for a given value of logarithmic measured ID_50_.

As illustrated in Figure 6, a regression analysis was conducted using standard antibodies for HPV6, HPV18, HPV33, HPV45, HPV52, and HPV58 produced by China National Institutes for Food and Drug Control. The logarithmic values of the theoretical ID_50_ (75%, 50%, and 25% diluted samples) were set as independent variables, while the logarithmic values of the corresponding observed ID_50_ served as dependent variables. The regression lines demonstrated strong linearity across all HPV types. Further evaluation of the linear correlation coefficients of HPV6, HPV18, HPV33, HPV45, HPV52, and HPV58 were from 0.983 to 1.000. All correlation coefficients met the predefined acceptance criterion (r ≥ 0.90), confirming a robust correlation between theoretical predictions and experimental measurements across samples with varying relative potency levels. These results collectively validate the high accuracy of the high-throughput detection method employed in this study for quantitative analysis of HPV antibody titers.

#### 3.4.4. Repeatability

To determine the robustness of the method, three operators were used to detect the 15-valent post-vaccination rabbit serum sample; each operator was repeatedly run three times and a total of 10 times each test for each sample. The intra-assay variability among different operators of positive serum was 10.9–19.4%, and the inter-assay variability was 13.4–33.1% (Table 2). All results satisfied the acceptance criteria (CV ≤ 50%), confirming the repeatability of this method.

#### 3.4.5. Cell Passages

As an important material for PBNA, cell culture is of great significance in biological assays involving pseudotyped virus infections. In order to investigate whether different cell passages have an impact on the results, neutralizing antibody detection was performed on the 293FT cells from the P22, P25, P28, and P31 generations. When the cell passage was within P31 generations, the GCV of different cell passages was 7.0% to 46.0% (Table 2). From the above results, there was no significant difference in antibody titers when cells were passaged within the range of P22 to P31.

#### 3.4.6. Z-Factor

Z-factor is a statistical parameter used to validate the robustness and applicability of high-throughput screening (HTS) experiments [19,20]. To determine whether the method is suitable for high-throughput screening, a negative control was added to half of a 384-well plate (152 wells), and a positive control was added (152 wells) in the other half. The Z-factor value of this method was 0.66 (95% CI 0.64–0.69), which indicated that it could be used as a reliable high-throughput neutralization test.

### 3.5. Comparison with Traditional 96-Well Plate Assay and High-Throughput 384-Well Plate Assay

Only four samples are allowed to be detected in one 96-well plate by using traditional PBNA, while 20 samples could be tested in one 384-well plate. Meanwhile, the volume of the reaction system, which was 200 μL in the 96-well plate assay, was reduced to 40 μL in the 384-well plate assay, resulting in a 5-fold reduction in the use of samples and pseudotyped viruses. Considering the efficiency and time required for detection, the 96-well plate assay can detect 120 15-valent samples per day, whereas the 384-well plate assay can detect up to 800 samples; thus, the detection throughput was increased by 6.7 times. The enhanced detection throughput achieved with the 384-well plate assay can be attributed to several key factors: the skipping of the pre-seeding step for cells, the cancellation of pseudovirus-serum neutralization time, and the synergistic use of a semi-automated liquid sample handling workstation along with an automated plate reader.

**Figure 7:**
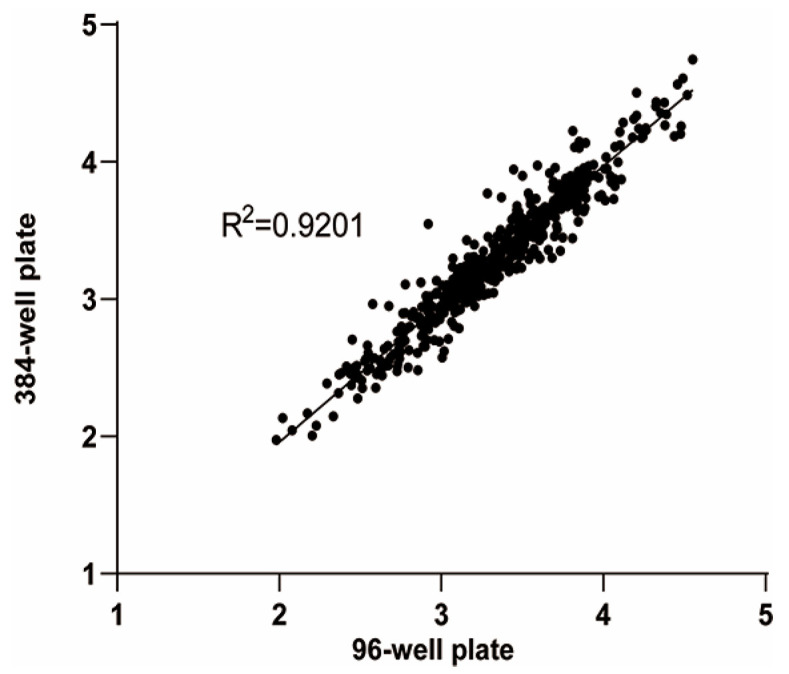
Comparison between traditional 96-well plate and high-throughput 384-well plate PBNA detection. The ID_50_ values were obtained by analyzing 57 human serum samples after vaccination with the nonavalent HPV vaccine using the two methods. The horizontal axis shows the logarithmic ID_50_ analyzed by the 96-well plate method, while the vertical axis indicates the logarithmic ID_50_ analyzed by the 384-well plate method.

To assess the consistency of fluorescence-positive cell numbers between the two methods, each sample immunized with nonavalent vaccine was processed at six different dilution gradients. Positive cell numbers obtained by both methods were then compared at identical dilutions. The Pearson correlation coefficient indicated a strong positive correlation, with r ≥ 0.90 (range 0.925–0.992) (Table 3). Furthermore, 57 human serum samples collected after vaccination with the nonavalent HPV vaccine were tested separately. There was no statistically significant difference in the ID_50_ values of each type between the two methods (*p* > 0.05), and the log-transformed ID_50_ of two methods correlated well, with R^2^ ≥ 0.90 (Figure 7).

## 4. Discussion

Cervical cancer remains a major cause of cancer incidence and mortality among women worldwide. Vaccination is the cornerstone of primary prevention, yet the evaluation of next-generation multivalent vaccines requires scalable, functional immunogenicity assays. Although a definitive serological correlate of protection for HPV has not been established, pseudovirion-based neutralization assays (PBNA) are widely used to quantify neutralizing antibodies. Traditional PBNA formats, however, are labor-intensive, time-consuming, and limited in throughput.

Here, we describe an automated 384-well PBNA that uses triple-color pseudotyped viruses to measure neutralizing antibodies against 15 HPV types. By selecting non-interfering type triplets based on cross-neutralization profiles and integrating semi-automated liquid handling with automated fluorescence readout, the assay reduces sample volume and assay duration while preserving concordance with the conventional 96-well format. We also establish serostatus cutoffs in an age-appropriate population, facilitating interpretation in clinical trials and vaccine development. The strengths of this approach include multiplex detection of three types per well without measurable interference, analytical validation across specificity, accuracy, precision, linearity, and robustness, and operational scalability suitable for large studies. Remaining considerations include the absence of an accepted correlate of protection, the need for harmonization to external standards and cross-laboratory comparability, potential residual interference among closely related types, and the possibility that serostatus cutoffs require recalibration in other populations.

Our lab previously developed a PBNA based on Gluc [15], which is as sensitive as SEAP-based PBNA but with faster detection (90 min to less than 5 min for a 96-well plate). However, the Gluc-PBNA is based on chemiluminescence, which requires additional reaction substrates during detection, resulting in relatively high costs. To address this, we developed a multi-color PBNA using fluorescent proteins [16]. This method utilizes pseudotyped viruses containing a variety of fluorescent reporter genes to simultaneously detect neutralizing antibodies against different types of HPV in a single well of the 96-well plate.

Importantly, the fluorescent proteins in the same combination should not interfere with each other under different excitation and emission light conditions. After comparison, green fluorescent protein (480/520 nm), red fluorescent protein (570/600 nm), and deep red fluorescent protein (630/670 nm) were selected as the combination for simultaneous detection. In this optimization study, the same reporter gene combination was used for high-throughput PBNA detection; due to the sequence similarity of HPV L1 within the same genus, the viruses that are distant relatives are preferred as a group when detecting multi-type antibodies to avoid interactions between different types [17]. For example, the pseudotyped virus combinations detected by the nonavalent vaccine can be divided into HPV16-18-58, HPV6-33-45, and HPV11-31-52 [16]. Meanwhile, the pseudotyped virus-infected cell counts are measured with FluoroSpot counting to calculate the titers of three types of neutralizing antibodies.

The cross-neutralization among various HPV types is a crucial factor in the classification of multi-type pseudotyped virus particle combinations. High-titer type-specific antibodies can be generated after vaccination with HPV types, and those antibodies also can simultaneously cross-neutralize the virus of another HPV type to varying degrees in vitro [21,22,23]. Therefore, the triple-color pseudotyped virus with cross-neutralizing effects should not be put into a combination, which can avoid subnormal levels of type-specific antibodies. In our study, the cross-neutralization effects among fifteen HPV types were investigated comprehensively. Some monovalent sera could cross-neutralize pseudotyped viruses of other types, such as HPV6/11 (sera/virus), HPV31/16, HPV58/33, HPV59/39, HPV18/45, HPV59/68, HPV59/16. This effect was consistent in sera from more than two animals in the same group. To meet the detection requirements of 15-valent serum samples, we retained the combination of HPV6-33-45 based on the study previously, also replaced HPV31-11-52 with HPV 31-11-58, and HPV16-18-52 with HPV16-18-68 [16]. Furthermore, the combination of HPV39-51-35 and HPV59-56-52 was added to an assay. In a previous study, Gao et al. [24] also validated a triple-color PBNA of 96-well plates for a 14-valent recombinant HPV vaccine, and 14 HPV viruses were divided into five groups to detect neutralizing antibodies (6-33-45, 31-11-58, 16-18-52, 39-51-35, 59-56). Although cross-reactivity was assessed, potential residual interference among closely related types cannot be entirely ruled out. Further investigation using more type-specific assays may be required to confirm these findings.

The cutoff value serves as a critical benchmark for determining the positivity of serum samples. To set the cutoff values, it is essential to assess the baseline serum titers values among individuals who will receive the vaccine in the future. In this study, we have successfully determined effective serostatus cutoff values (mean + 3 SD). The defined cutoff titers against HPV 6/11/16/18/31/33/35/39/45/51/52/56/58/59/68 were as follows: 177, 97, 85, 100, 77, 112, 82, 48, 148, 52, 84, 130, 125, 97, and 68. In a clinical trial targeting the nonavalent human papillomavirus L1 VLPs vaccine, the study used the threshold established in this research as the cutoff values for neutralizing antibodies against HPV. The results showed that the baseline seropositive rates of HPV 16 (4.5%), HPV 18 (1.4%), HPV 45 (0.6%), and HPV 52 (3.2%) were consistent with the prevalence rates reported in previous studies in China [25]. Additionally, the seropositive rate of low-risk HPV6 was 3.8%, which is similar to the prevalence rate reported in a nationwide survey of HPV6 (4.01%) infections in Chinese women [26]. However, the serostatus cutoffs used to define seropositive status are derived from a specific cohort of this study. Therefore, their applicability to different populations (such as different age groups or geographic regions) may require reevaluation and calibration.

We developed an automated, high-throughput PBNA in a 384-well format to assess immunogenicity across 15 HPV types relevant to multivalent vaccine development. Replacing 96-well plates with 384-well plates and integrating semi-automated liquid-handling workstations improved operational stability and expanded type coverage. Guided by cross-neutralization profiling, the 15 pseudotyped viruses were partitioned into five triple-color combinations, enabling simultaneous detection of three fluorescence signals per well and thereby shortening assay time. Automated imaging on the Cytation 5 allows precise quantification of cells expressing distinct fluorescent proteins; combined with a microplate stacker, the workflow supports unattended (“lights-out”) runs. Compared with the conventional 96-well method, daily sample throughput increased ~6.7-fold, and for an equivalent sample load the imaging time decreased ~4-fold (from ~75 to ~19 min versus the ELISPOT ImmunoSpot reader). Z-factor analysis further supports the suitability of this platform for high-throughput applications.

## 5. Conclusions

We optimized and validated an automated pseudovirion-based neutralization assay in a 384-well format that quantifies neutralizing antibodies against 15 HPV types using triple-color readouts. This platform enables scalable, multiplex functional immunogenicity assessment for current and next-generation multivalent HPV vaccines and is suitable for large clinical studies and seroepidemiology. Future work should prioritize cross-laboratory harmonization to international standards and evaluate how neutralization titers relate to protection across diverse populations.

## Figures and Tables

**Table 1:** The groups of triple-color pseudotyped virus.

Fluorescent Protein	GFP	RFP	CFP
Group 1	HPV6	HPV33	HPV45
Group 2	HPV31	HPV11	HPV58
Group 3	HPV16	HPV18	HPV68
Group 4	HPV39	HPV51	HPV35
Group 5	HPV59	HPV56	HPV52

**Table 2:** Establishment and validation parameter summary table.

Characteristic	HPV Types														
	HPV6	HPV11	HPV16	HPV18	HPV31	HPV33	HPV35	HPV39	HPV45	HPV51	HPV52	HPV56	HPV58	HPV59	HPV68
Serostatus cutoff (ID_50_)	177	97	85	100	77	112	82	48	148	52	84	130	125	97	68
Positive rate (%)	1.4	2.0	3.6	2.9	3.4	1.3	1.7	1.0	1.2	2.2	3.0	2.7	1.5	2.3	1.0
Intra-assay precision (CV%)	16.3	14.5	21.4	13.7	14.9	10.9	12.6	16.8	12.3	11.5	14.2	16.0	12.4	19.4	13.9
Inter-assay precision (CV%)	24.1	19.6	27.8	18.3	24.0	21.5	14.3	17.1	23.7	13.4	21.1	23.3	26.4	33.1	22.0
Cell concentration (GCV%)															
750 cells/well	26.1	9.4	12.5	16.7	6.2	11.1	7.9	4.3	9.6	7.6	7.4	7.6	9.5	8.0	13.8
1500 cells/well	14.4	6.5	17.1	9.5	9.4	7.5	8.1	7.2	6.9	13.6	4.8	10.6	12.1	8.9	7.7
3000 cells/well	31.6	12.6	40.4	17.5	22.7	23.0	23.5	24.1	20.6	19.5	17.2	10.9	27.3	19.3	25.5
6000 cells/well	39.1	20.4	60.4	31.3	46.6	23.1	22.1	22.3	24.2	13.7	20.9	17.6	24.2	29.8	43.9
12,000 cells/well	49.3	31.1	119.4	27.3	43.9	29.2	24.0	20.4	30.7	40.0	33.7	42.9	39.8	46.7	59.1
Virus-serum neutralization time (GCV%)															
0 h	15.0	6.8	39.2	34.3	21.0	14.1	7.5	3.6	15.1	7.6	10.7	11.7	29.1	9.7	33.9
0.5 h	12.3	9.9	46.7	35.9	7.8	16.7	7.1	4.3	15.2	6.3	13.6	16.1	24.1	20.1	44.1
1 h	29.4	6.4	17.1	13.1	19.7	13.5	10.5	8.3	13.4	7.7	11.9	15.2	23.1	16.4	9.1
2 h	11.4	6.5	10.3	16.0	12.4	16.3	10.6	6.4	12.8	10.2	8.6	13.1	24.9	16.2	21.2
Incubation time (GCV%)															
48 h	22.0	39.6	35.7	29.8	39.8	51.0	180.7	29.2	29.4	27.9	42.6	39.6	112.4	13.6	12.2
60 h	29.1	24.1	19.6	18.0	26.6	30.3	24.5	28.0	28.0	19.6	22.4	15.8	38.2	9.1	28.9
72 h	24.1	22.3	19.8	22.8	21.5	26.5	28.5	19.4	30.6	20.3	15.4	12.8	43.8	5.2	22.0
96 h	36.0	23.8	21.6	20.0	21.4	25.9	22.2	16.7	26.6	20.4	15.7	11.4	40.5	8.7	18.5
120 h	50.3	24.5	17.7	27.2	36.1	34.6	21.8	22.0	39.4	26.8	11.1	12.6	45.0	9.4	16.2
Cell passages (GCV%)															
P22	23.6	16.4	24.2	13.5	12.7	10.4	10.2	13.5	10.7	11.5	10.4	16.1	9.0	15.9	15.2
P25	11.1	15.7	46.0	23.6	10.4	12.1	22.9	19.1	8.3	16.5	9.5	11.4	11.0	10.8	20.6
P28	12.5	11.8	19.6	13.3	10.5	8.9	17.5	17.5	8.2	11.5	7.0	12.1	15.6	12.3	18.7
P31	15.6	10.5	21.6	12.8	12.4	9.1	12.2	11.7	9.5	8.7	8.4	13.4	12.8	11.6	14.7

**Table 3:** The correlation of fluorescent positive cell numbers between the 96-well plate assay and the 384-well plate assay.

Pseudotyped HPV	Pearson’s Correlation Coefficient (r)
HPV6-EGFP	0.985
HPV16-EGFP	0.992
HPV31-EGFP	0.988
HPV11-RFP	0.986
HPV18-RFP	0.984
HPV33-RFP	0.987
HPV45-CFP	0.925
HPV52-CFP	0.932
HPV58-CFP	0.989

## Data Availability

All data supporting this study are included in the article and its supplementary materials.

## Supplementary Materials

The following supporting information can be downloaded at: https://www.mdpi.com/article/10.3390/v17091164/s1, Figure S1: Specificity: rabbit serum immunized with a 15-valent vaccine was subjected to neutralization tests against irrelevant pseudotyped virus (SARS-CoV-2) (A). Negative serum (B) and irrelevant monoclonal antibodies (SARS-CoV-2) (C) were used as controls in the neutralization tests against the pseudotyped virus of SARS-CoV-2. The horizontal axis shows the logarithmic dilutions of sera, while the vertical axis indicates the corresponding inhibition rates (%); Table S1: The ID50 values of type specific antibodies and cross-reactive antibodies obtained from single positive rabbit serum detected by different HPV pseudotyped viruses (N = 45); Table S2: The analysis of correlation and ANOVA between triple-type and single-type pseudotyped virus detection assays.

## References

[1] Bray F., Laversanne M., Sung H., Ferlay J., Siegel R.L., Soerjomataram I., Jemal A. (2024). Global cancer statistics 2022: GLOBOCAN estimates of incidence and mortality worldwide for 36 cancers in 185 countries. CA Cancer J. Clin..

[2] Mühr L.S.A., Eklund C., Dillner J. (2018). Towards quality and order in human papillomavirus research. Virology.

[3] De Villiers E.M. (2013). Cross-roads in the classification of papillomaviruses. Virology.

[4] International Human Papillomavirus Reference Center (2023). HPV Reference Clones. https://www.hpvcenter.se/human_reference_clones/.

[5] Scarth J.A., Patterson M.R., Morgan E.L., Macdonald A. (2021). The human papillomavirus oncoproteins: A review of the host pathways targeted on the road to transformation. J. Gen. Virol..

[6] Williamson A.L. (2023). Recent Developments in Human Papillomavirus (HPV) Vaccinology. Viruses.

[7] Zhang X., Meng D., Li H., Li X., Li J., Hu P., Zhao L., Wang R., Zhao C., Luo C. (2023). Validation of Luminex immunological and competitive Luminex immunological assays for clinical immunogenicity assessment of a 14-valent recombinant human papillomavirus vaccine. J. Med. Virol..

[8] Villa L.L., Costa R.L., Petta C.A., Andrade R.P., Ault K.A., Giuliano A.R., Wheeler C.M., Koutsky L.A., Malm C., Lehtinen M. (2005). Prophylactic quadrivalent human papillomavirus (types 6, 11, 16, and 18) L1 virus-like particle vaccine in young women: A randomised double-blind placebo-controlled multicentre phase II efficacy trial. Lancet Oncol..

[9] Mao C., Koutsky L.A., Ault K.A., Wheeler C.M., Brown D.R., Wiley D.J., Alvarez F.B., Bautista O.M., Jansen K.U., Barr E. (2006). Efficacy of human papillomavirus-16 vaccine to prevent cervical intraepithelial neoplasia: A randomized controlled trial. Obstet. Gynecol..

[10] WHO (2009). Human Papillomavirus Laboratory Manual.

[11] Buck C.B., Pastrana D.V., Lowy D.R., Schiller J.T. (2004). Efficient intracellular assembly of papillomaviral vectors. J. Virol..

[12] Buck C.B., Pastrana D.V., Lowy D.R., Schiller J.T. (2005). Generation of HPV pseudovirions using transfection and their use in neutralization assays. Methods Protoc..

[13] Pastrana D.V., Buck C.B., Pang Y.Y., Thompson C.D., Castle P.E., FitzGerald P.C., Kjaer S.K., Lowy D.R., Schiller J.T. (2004). Reactivity of human sera in a sensitive, high-throughput pseudovirus-based papillomavirus neutralization assay for HPV16 and HPV18. Virology.

[14] Sehr P., Rubio I., Seitz H., Putzker K., Ribeiro-Müller L., Pawlita M., Müller M. (2013). High-throughput pseudovirion-based neutralization assay for analysis of natural and vaccine-induced antibodies against human papillomaviruses. PLoS ONE.

[15] Nie J., Huang W., Wu X., Wang Y. (2014). Optimization and validation of a high throughput method for detecting neutralizing antibodies against human papillomavirus (HPV) based on pseudovirons. J. Med Virol..

[16] Nie J., Liu Y., Huang W., Wang Y. (2016). Development of a Triple-Color Pseudovirion-Based Assay to Detect Neutralizing Antibodies against Human Papillomavirus. Viruses.

[17] De Vincenzo R., Ricci C., Conte C., Scambia G. (2013). HPV vaccine cross-protection: Highlights on additional clinical benefit. Gynecol. Oncol..

[18] Wu X., Nie J., Wang Y. (2023). Pseudotyped Virus for Papillomavirus. Adv. Exp. Med. Biol..

[19] Martins Lima A., Bragina M.E., Burri O., Bortoli Chapalay J., Costa-Fraga F.P., Chambon M., Fraga-Silva R.A., Stergiopulos N. (2019). An optimized and validated 384-well plate assay to test platelet function in a high-throughput screening format. Platelets.

[20] Zhang J.H., Chung T.D., Oldenburg K.R. (1999). A Simple Statistical Parameter for Use in Evaluation and Validation of High Throughput Screening Assays. J. Biomol. Screen..

[21] Romanowski B., de Borba P.C., Naud P.S., Roteli-Martins C.M., De Carvalho N.S., Teixeira J.C., Aoki F., Ramjattan B., Shier R.M., GlaxoSmithKline Vaccine HPV-007 Study Group (2009). Sustained efficacy and immunogenicity of the human papillomavirus (HPV)-16/18 AS04-adjuvanted vaccine: Analysis of a randomised placebo-controlled trial up to 6.4 years. Lancet.

[22] Kavanagh K., Pollock K.G., Cuschieri K., Palmer T., Cameron R.L., Watt C., Bhatia R., Moore C., Cubie H., Cruickshank M. (2017). Changes in the prevalence of human papillomavirus following a national bivalent human papillomavirus vaccination programme in Scotland: A 7-year cross-sectional study. Lancet Infect. Dis..

[23] Hoes J., King A.J., Berkhof J., de Melker H.E. (2023). High vaccine effectiveness persists for ten years after HPV16/18 vaccination among young Dutch women. Vaccine.

[24] Gao S., Zhao D., Feng C., Kou Y., Lu J., Luo C., Li X., Wang Y., Xie L. (2024). Validation of a triple-color pseudovirion-based neutralization assay for immunogenicity assessment of a 14-valent recombinant human papillomavirus vaccine. J. Med. Virol..

[25] Zeng Z., Austin R.M., Wang L., Guo X., Zeng Q., Zheng B., Zhao C. (2022). Nationwide Prevalence and Genotype Distribution of High-Risk Human Papillomavirus Infection in China. Am. J. Clin. Pathol..

[26] Wang R., Guo X.L., Wisman G.B., Schuuring E., Wang W.F., Zeng Z.Y., Zhu H., Wu S.W. (2015). Nationwide prevalence of human papillomavirus infection and viral genotype distribution in 37 cities in China. BMC Infect. Dis..

