# Supplementary material for: Optimization of a High-Throughput Human Papillomavirus Neutralizing Antibody Assay Based on Pseudotyped Viruses for the 15-Valent Human Papillomavirus Vaccine Types
Source: Viruses. 2025 Aug 26;17(9):1164. doi: 10.3390/v17091164 (PMC12474018; doi:10.3390/v17091164)
Supplement: Supplementary file 1 [file viruses-17-01164-s001.zip › viruses-3827288-supplementary.pdf]

Tabel S1. The ID50 values of type specific antibodies and cross-reactive antibodies obtained from single positive rabbit serum detected by different HPV pseudotyped viruses (N=45)

Rabbit	Psedotyped virus														
	HPV6	HPV11	HPV16	HPV18	HPV31	HPV33	HPV35	HPV39	HPV45	HPV51	HPV52	HPV56	HPV58	HPV59	HPV68
HPV6-R1	231953	6348	<400	<400	<400	<400	<400	<400	<400	<400	<400	<400	<400	<400	<400
HPV6-R2	376871	3858	<400	<400	<400	<400	<400	<400	<400	<400	<400	<400	<400	<400	<400
HPV6-R3	743310	15823	<400	<400	<400	<400	<400	<400	<400	<400	<400	<400	<400	<400	<400
HPV11-R4	12960	59621	<400	<400	<400	<400	<400	<400	<400	<400	<400	<400	<400	<400	<400
HPV11-R5	43001	19610	<400	<400	<400	<400	<400	<400	<400	<400	<400	<400	<400	<400	<400
HPV11-R6	6372	34580	<400	<400	<400	<400	<400	<400	<400	<400	<400	<400	<400	<400	<400
HPV16-R7	<400	<400	71384	<400	452	<400	<400	<400	<400	<400	<400	<400	<400	710	<400
HPV16-R8	<400	<400	69898	<400	659	<400	<400	<400	<400	<400	<400	<400	1278	<400	<400
HPV16-R9	<400	<400	131215	<400	1240	<400	<400	<400	<400	<400	<400	<400	<400	493	<400
HPV18-R10	<400	<400	<400	85981	<400	<400	<400	<400	<400	<400	<400	<400	<400	<400	<400
HPV18-R11	<400	<400	<400	114039	<400	<400	<400	<400	<400	<400	<400	<400	<400	<400	<400
HPV18-R12	<400	<400	<400	202574	<400	<400	<400	<400	<400	<400	<400	<400	<400	<400	<400
HPV31-R13	<400	<400	<400	<400	391145	<400	<400	<400	<400	<400	5810	<400	<400	<400	<400
HPV31-R14	<400	<400	<400	<400	99082	<400	<400	<400	<400	<400	<400	<400	<400	<400	<400
HPV31-R15	<400	<400	<400	<400	92072	<400	<400	<400	<400	<400	<400	<400	<400	<400	<400
HPV33-R16	<400	<400	<400	<400	<400	54251	<400	<400	<400	<400	<400	<400	17119	764	<400
HPV33-R17	<400	<400	<400	<400	<400	15858	<400	<400	<400	<400	<400	<400	3004	<400	<400
HPV33-R18	<400	<400	<400	<400	<400	64656	<400	<400	<400	<400	<400	<400	30648	<400	<400
HPV35-R19	<400	<400	<400	<400	<400	<400	44407	<400	<400	<400	<400	<400	<400	<400	<400
HPV35-R20	<400	<400	<400	<400	<400	<400	31391	<400	<400	<400	<400	<400	<400	<400	<400
HPV35-R21	<400	<400	<400	<400	<400	<400	17817	<400	<400	<400	<400	<400	<400	<400	<400
HPV39-R22	<400	<400	<400	<400	<400	<400	<400	8001	<400	<400	<400	<400	<400	1841	<400
HPV39-R23	<400	<400	<400	<400	<400	<400	1374	31831	<400	<400	<400	<400	<400	2152	1044
HPV39-R24	<400	<400	<400	<400	<400	<400	<400	5445	<400	<400	<400	<400	<400	1207	<400

HPV45-R25	<400	<400	<400	3862	<400	<400	<400	<400	86468	<400	<400	<400	<400	<400	<400
HPV45-R26	<400	<400	<400	3313	<400	<400	<400	<400	105987	<400	<400	<400	<400	<400	<400
HPV45-R27	<400	<400	<400	930	<400	<400	<400	<400	6035	<400	<400	<400	<400	<400	<400
HPV51-R28	<400	<400	<400	<400	<400	<400	<400	<400	<400	52194	<400	<400	<400	<400	<400
HPV51-R29	<400	<400	<400	<400	<400	<400	<400	<400	<400	23744	<400	<400	<400	<400	<400
HPV51-R30	<400	<400	<400	<400	<400	<400	<400	<400	<400	81993	<400	<400	<400	<400	<400
HPV52-R31	<400	<400	<400	<400	<400	<400	<400	<400	<400	<400	573251	<400	<400	<400	<400
HPV52-R32	<400	<400	<400	<400	<400	<400	<400	<400	<400	<400	111542	<400	<400	<400	<400
HPV52-R33	<400	<400	<400	<400	<400	1288	<400	<400	<400	<400	83102	<400	470	<400	<400
HPV56-R34	<400	<400	<400	<400	<400	<400	<400	<400	<400	<400	<400	182209	<400	611	<400
HPV56-R35	<400	<400	573	<400	<400	<400	<400	<400	<400	<400	<400	274291	<400	<400	<400
HPV56-R36	<400	<400	<400	<400	<400	<400	<400	<400	<400	<400	<400	301909	<400	<400	<400
HPV58-R37	<400	<400	<400	<400	<400	<400	<400	<400	<400	<400	<400	<400	60957	<400	<400
HPV58-R38	<400	<400	<400	<400	<400	2983	<400	<400	<400	<400	<400	<400	57619	<400	<400
HPV58-R39	<400	<400	<400	<400	<400	1894	<400	<400	<400	<400	<400	<400	158316	<400	<400
HPV59-R40	<400	<400	<400	<400	<400	<400	<400	945	<400	<400	<400	<400	<400	67841	<400
HPV59-R41	<400	<400	<400	<400	<400	<400	<400	672	<400	<400	<400	<400	<400	161740	<400
HPV59-R42	<400	<400	<400	<400	<400	<400	<400	849	<400	<400	<400	<400	<400	159609	<400
HPV68-R43	<400	<400	<400	<400	<400	<400	<400	752	<400	<400	<400	<400	<400	464	37023
HPV68-R44	<400	<400	<400	2865	<400	<400	<400	<400	<400	<400	<400	<400	<400	638	38467
HPV68-R45	<400	<400	<400	<400	<400	<400	<400	<400	<400	<400	<400	<400	<400	489	18453

Table S2. The analysis of correlation and ANOVA between triple-type and single-type pseudotyped viruses detection assays

Groups	Psedotyped types	triple-type mean	single-type mean	triple-type standard deviation	single-type standard deviation	Correlation analysis r	Correlation analysis *p* value	ANOVA F	ANOVA *p* value
Group A	HPV6	41924	52289	21367	28785	0.95	<0.001	1.51	0.23
	HPV33	10480	9985	5840	5219	0.99	<0.001	0.07	0.79
	HPV45	11055	9848	4467	4118	0.98	<0.001	0.71	0.41
Group B	HPV31	5534	5301	1650	1599	0.90	<0.001	0.19	0.67
	HPV11	5825	5935	3113	3685	0.99	<0.001	0.01	0.92
	HPV58	38344	35441	14965	13752	0.97	<0.001	0.37	0.55
Group C	HPV16	61392	67130	30714	36996	0.98	<0.001	0.26	0.62
	HPV18	44651	50635	16370	22182	0.92	<0.001	0.83	0.37
	HPV68	28540	32774	20581	28316	0.97	<0.001	0.26	0.61
Group D	HPV39	20348	24897	11561	14928	0.93	<0.001	1.05	0.31
	HPV51	10701	12435	4538	5613	0.96	<0.001	1.04	0.32
	HPV35	9122	10330	3423	3835	0.96	<0.001	0.99	0.33
Group E	HPV59	36461	50246	18860	28042	0.95	<0.001	3.00	0.09
	HPV56	48546	58974	39718	50367	0.98	<0.001	0.48	0.5
	HPV52	16011	11927	11086	8489	0.99	<0.001	1.54	0.22

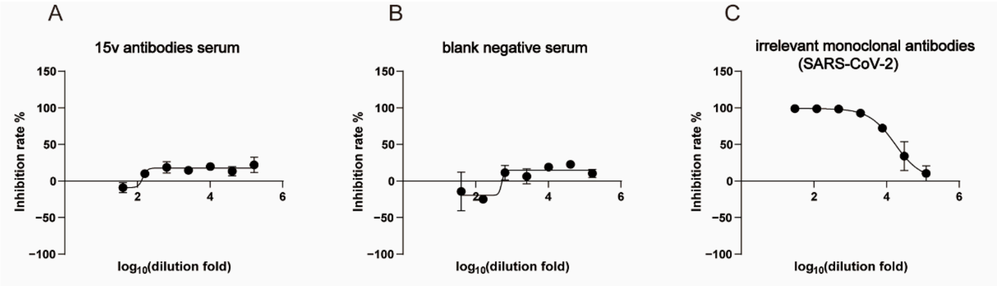

Figure S1. Specificity: rabbit serum immunized with a 15-valent vaccine was subjected to neutralization tests against irrelevant pseudotyped virus (SARS-CoV-2) (A). Negative serum (B) and irrelevant monoclonal antibodies (SARS-CoV-2) (C) were used as controls in the neutralization tests against the pseudotyped virus of SARS-CoV-2. The horizontal axis shows the logarithmic dilutions of sera, while the vertical axis indicates the corresponding inhibition rates (%).
